# Executive functions and information processing in patients with type 2 diabetes in comparison to pre-diabetic patients

**DOI:** 10.1186/2251-6581-13-27

**Published:** 2014-02-04

**Authors:** Marzieh Nazaribadie, Masoud Amini, Mohammad Ahmadpanah, Karim Asgari, Somaye Jamlipaghale, Sara Nazaribadie

**Affiliations:** 1 Clinical Psychology Ward, Farshchian Hospital, Hamadan University of Medical Sciences, Hamadan, Iran; 2 Isfahan Endocrine and Metabolism Research Center, Isfahan University of Medical Sciences, Isfahan, Iran; 3 Research Center for Behavioral Disorders and Substances Abuse, Hamadan University of Medical Sciences, Hamadan, Iran; 4 Department of Psychology, School of Psychology and Educational Sciences, University of Isfahan, Isfahan, Iran; 5 Department of Psychology, School of Human Sciences, Alzahra University, Tehran, Iran; 6 Department of Nursing, School of Nursing and Midwifery, Hamadan University of Medical Sciences, Hamadan, Iran

**Keywords:** Executive functions, Information processing, Type 2 Diabetes, Pre-diabetic patients

## Abstract

**Background:**

Diabetes is associated with cognitive decline or dementia. The purpose of this study was to assess the executive functions and information processing in patients with type 2diabetes in comparison to pre-diabetic patients and normal subjects in Endocrine and Metabolism Research Center of Isfahan City from April to July 2011.

**Methods:**

The sample consisted of 32 patients with type 2 diabetes, 28 pre-diabetic patients and 30 healthy individuals. Executive functions were assessed by Wisconsin Card Sorting Test (WCST). Information processing was assessed by Paced Auditory Serial Addition Test (PASAT) and sub tests of Wechsler Adult Intelligence Scale-Revised (WAIS-R).

**Results:**

There was a significant difference among 3 groups, after the variables of age, sex and academic status were controlled (p ≤ 0.001). The pairwise comparisons of executive functions among three groups suggest a significant difference between diabetic and normal groups in WCST (perseveration) p = 0.018, and significant difference between diabetic and pre-diabetic patient in WCST (perseveration) p = 0.019. But there was no difference between three groups in WCST (category) and WCST (conceptual responses). The pairwise comparisons of information processing among three groups, suggest a significant difference between diabetic and normal groups in PASAT3". PASAT2", and Symbol coding (*P* = 0.003, *P* = 0.009, and *P* = 0.001, respectively). There was a significant correlation between demographic variable (FBS, HbA1c) and Symbol coding p = 0.05, p = 0.01 respectively) and significant correlation between (cholesterol) and WCST (conceptual responses) p = 0.05. The other variables were not correlated.

**Conclusion:**

There were significant differences in executive function and information processing in patients with type 2 diabetic and normal individuals. Thus, monitoring neuropsychological status besides controlling levels of blood sugar in these patients is important.

## Introduction

Several reports have indicated that diabetes may cause cognitive dysfunction [1-3] or an alternation in brain signals related to cognitive function [4-6] and accelerated cognitive decline [7,8]. Recent epidemiological studies suggest that diabetes mellitus is a stronger risk factor for Alzheimer disease [9-13]. Patient with diabetes mellitus show increased progression of brain atrophy [2,14]. Several research studies following large groups over many years suggest that adults with type 2 diabetes have a higher risk of later developing Alzheimer′s, and the risk effects are stronger when diabetes occurs mid life than in late life [15]. Previous studies have shown decrement in executive functions and information processing [16-18]. The stage of cognitive decrements is not manifested and may occur in pre- diabetic stage. Pre-diabetes period is the stage of impaired glucose regulation, or blood sugar levels that are higher than normal but not yet in the diabetic range. Insulin resistance (pre-diabetes period) may be maker of Alzheimer disease associated with reduced cognitive impairment at the earliest of disease, even before the onset of mild cognitive impairment [6,11,13,19]. Glycemic control appears to play an important role in preserving cognitive performance among patient with type 2 diabetes. In patients with type 2 diabetes, studies have demonstrated an inverse relationship between serum Hb1Ac and working memory, executive functioning, learning and complex psychomotor performance. This finding supports the hypothesis that an inadequate glucose control is associated with worsening cognitive function [19]. The few studies that have been conducted on pre-diabetic period [18] and also a study performed on the patients indicated that patients in pre-diabetic period experience decline in memory functioning [10]. But there was no significant difference in other types of performance such as attention and visual-spatial function [10]. According to the importance of the variable in studies and the fact that some functions such as executive function and information processing are not measured in this study, we intend to investigate executive and information processing functioning in diabetic and pre-diabetic patients.

## Methods

This study was a cross-sectional one which was started in April 2011 and ended in July 2011 in Endocrine and Metabolism center of Isfahan city. Sample size was 32 for diabetes, 28 for pre-diabetes, and 30 for normal subjects. Diabetic and pre-diabetic patients were selected after the diagnosis made by a specialist according to American Diabetes Association criteria for diagnosis, and also according to their clinical data recorded in their files. Correlation coefficients between the research variables and the demographic variables including age, sex and academic status were controlled by the researcher. The inclusion criteria for entering the study were: being diabetic or pre-diabetic according to the diagnosis made by a specialist, age range between 35 to 60 years, being educated (from grade 9 and up). Clients had to have normal or corrected vision and hearing sufficient to adequately comprehend the test instruction and to discriminate visually the stimulus parameters of color, form, number and finally have no depression. Psychiatric interview was conducted according to DSM-TV-R for screening depression by MSc in clinical psychology. The control group was matched with the experimental groups, and selected from the personnel of University of Isfahan, and from personnel of some elementary schools. The patients were selected randomly according to the research criteria. The criteria for diagnosis of diabetes according to American Diabetes Association is fasting plasma glucose (FPG) at or above 126 mg/dL (7.0 mmol/L), at 2-h value in an oral tolerance test (OGGT) at or above 200 mg/dL (11/1 mmol/L) and if plasma glucose test is greater than 126 mg/dL, one might have impaired fasting glucose (IFG). Some people also have impaired glucose tolerance (IGT), a condition in which blood glucose levels are higher than normal (140 mg/dL to 199 mg/dc) 2 hours after the start of an oral glucose tolerance test (GTT) if one has IFG and or IGT, one may be diagnosed with pre-diabetes American Diabetes Association, 2005 [20].

Written consent was taken from each patient, and they were interviewed by the researcher for not being clinically depressed. Then, all of the three groups were assessed by executive function tests. Other clinical and demographic data were obtained from each patient’s files.

### Executive functional assessment

Wisconsin Card Sorting Test (WCST): the purpose of this test is to assess the ability to form abstract concept, shift and maintain set, and utilize feedback. This test was designed to assess abstraction ability and the ability to shift cognitive strategies in response to changing environmental contingencies. The test is considered to measure executive function. The perseveration responses of this test reveal an ability to relinquish the old category for the new one, or the inability to see a new possibility. Category gives an indication of initial conceptualization, in turn, reflecting the concentration of perseveration errors in relation to overall test performance [21]. The interrater reliability of this test is 0.83 and test -retest of this test is 0.74 [22]. The reliability of this test has been reported to be about 0.85 [23].

### Information processing assessment

1. Tests of Symbol Coding from the Wechsler Adult Intelligence Scales-Revised (WAIS-R) test battery [24]. This sub test was used to assess information processing. Test –retest reliability of the test, is reported to be 0.90 [25].

2. Paced Auditory Serial Addition Test (PASAT). This test is a serial addition task used to assess rate information processing and attention [21]. The reliability of this test was 0.90. The reliability of the test was calculated by the authors of this article. The calculated Cronbach's alpha was 0.74.

### Statistical analysis

1. Shapiro - Wilk and Kolgomorov Smirnov tests were used to test normality for data, and in some data the Kruskal-Wallis test was used instead of parametric statistical tests. Also, Leven test was used in order to test equality of variances. There was equality of variances for the result of symbol coding, however, there haven't been seen equality of variances for Paced Auditory Serial Addition Test (PASAT), therefore, in addition to ANCOVA, the non parametric test was also done for the data.

2. The equality of variances was confirmed for the results of WCST (perseveration); however, there were no equality of variances for WCST (conceptual responses) and WCST (category), hence, in addition to ANCOVA, the non parametric tests were also applied to the data.

3. Since the authors have used analysis of covariance for the data, first of all the correlations between executive function, information processing and demographic variables (age, education and gender) were calculated, and then those variables with significant effects were controlled; and finally the analysis of covariance was used in order to find possible differences between executive function in three groups.

## Results

Table 1 shows median and standard deviation in demographic variables (education and age) in three groups diabetes, pre- diabetes and normal subjects. As well as shows median and standards deviation in clinical variables (HbA1c, FBS, 2hpp and cholesterol) in patients with diabetes and pre- diabetes groups. As can be seen, the mean age of diabetic group is more and greater than the mean age of control and pre diabetics, so we can concluded that diabetes may associate with aging. There were no significant differences in educational level of three groups.

**Table 1:** demographic and clinical characteristics of 3 groups

	**Group**	**Mean**	**Std. deviation**	**N**
Age	diabetes type 2	50.43	6.53	32
	Pre-diabetes	48.46	6.66	28
	Control group	44.93	6.10	30
Education	diabetes type 2	12.31	2.38	32
	Pre-diabetes	12.35	1.96	30
	Control group	12.74	2.38	28
HbA1c	diabetes type 2	7.51	2.01	30
	Pre-diabetes	5.26	0.55	28
FBS	diabetes type 2	165.0	84.17	30
	Pre-diabetes	110.9	14.56	28
2hpp	diabetes type 2	228.1	86.93	30
	Pre-diabetes	134.5	46.56	28
Cholesterol	diabetes type 2	172.3	35.87	30
	Pre-diabetes	183.9	50.49	28

According to Table 2, there was a significant difference among 3 groups, after the variables of age; sex and academic status (p ≤ 0.001) were controlled. Eta square shows that 19% of the difference in executive function can be due to the difference among three groups.

**Table 2:** Results of multivariate analysis of variance in patients with type 2 diabetes, pre-diabetes and control group

**Statistical index**	**Wilk lambda**	**F**	**Sig**	**Partial Eta squared**	**Observed power**
Age	0.87	1.88	0.09	0.12	0.66
Gender	0.77	3.91	0.002	0.22	0.95
Education	0.60	8.45	0.0001	0.39	1
Group	0.65	3.11	0.001	0.19	0.99

According to Table 3, there was a significant correlation between demographic variable (FBS, HbA1c) and Symbol coding (p = 0.05, p = 0.01 respectively) and significant correlation between (cholesterol) and WCST (conceptual responses) p = 0.05. The other variables were not correlated.

**Table 3:** Correlation coefficient and significant coefficient of research variables in 2 groups, diabetes type 2 and pre-diabetes

**Research variables**		**FBS**	**HbA1c**	**Cholesterol**
PASAT3"	Correlation	0.18	-0.20	0.16
	Sig	0.16	0.13	0.27
PASAT2"	Correlation	0.16	-0.14	0.20
	Sig	0.21	0.27	0.17
WCST (conceptual responses)	Correlation	-0.11	0.13	0.17*
	Sig	0.41	0.33	0.04
WCST (perseveration)	Correlation	0.20	0.12	-0.20
	Sig	0.88	0.36	0.30
WCST (category)	Correlation	0.20	0.12	-0.20
	Sig	0.88	0.36	0.30
Symbol coding	Correlation	0.25*	0.38**	0.05
	Sig	0.05	0.004	0.70

**Correlation is significant at the 0.01 level. *Correlation is significant at the 0.05 level.

According to Table 4, the difference in information processing among three groups was statistically significant for PASAT3" (*P* = 0.012), PASAT2" (*P* = 0.033 and symbol coding (*P* = 0.001). The difference in executive functions among three groups was statistically significant for WCST (perseveration) p = 0.025 but not for WCST p = 0.35 and WCST (conceptual responses) p = 0.28.

**Table 4:** Results of multivariate analysis of covariate in patients with type 2 diabetes, pre-diabetes and control group

**Independent variable**	**Variable**	**Sumof square**	**df**	**Sig**	**Partial Eta squared**	**Observed power**
**Group**	PASAT3"	705.78	2	0.01	0.10	0.77
	PASAT2"	399.62	2	0.03	0.07	0.64
	WCST (perseveration)	179.985	2	0.025	0.084	0.68
	WCST (category)	4.546	2	0.35	0.24	0.22
	Symbol coding	1865.52	2	0.0001	0.24	0.99
	WCST (conceptual responses)	15.274	2	0.25	0.032	0.28

Table 5 shows the pairwise comparisons of information processing among three groups, suggesting a significant difference between diabetic and normal groups in PASAT3". PASAT2", and Symbol coding (*P* = 0.003, *P* = 0.009, and *P* = 0.001, respectively). There was a significant difference between pre-diabetic group and normal group between diabetes type 2 and pre-diabetic group in symbol coding (*P* = 0.001), and the pairwise comparisons of executive functions among three groups, suggesting a significant difference between diabetic and normal groups in WCST (perseveration) p = 0.018, and significant difference between diabetic and pre-diabetic patient in WCST (perseveration) p = 0.019. But there was no difference between three groups in WCST (category) and WCST (conceptual responses).

**Table 5:** Results of pairwise comparisons in patients with type 2 diabetes, pre-diabetes and normal group

**Dependent variable**	**Groups**		**Mean**	**Std. error**	**Sig**
**WCST (perseveration)**	type 2 diabetes	Pre-diabetic	3.005	1.25	0.019
		Normal	3.146	1.30	0.018
	Pre-diabetes	Normal	0.141	1.301	0.914
**WCST (category)**	type 2 diabetes	Pre-diabetic	-0.360	0.386	0.55
		Normal	-0.566	0.401	0.162
	Pre-diabetes	Normal	-.206	0.399	0.608
**WCST (conceptual responses)**	type 2 diabetes	Pre-diabetic	-0.595	0.615	0.336
		Normal	-1.051	0.638	0.103
	Pre-diabetes	Normal	-.451	0.635	0.474
**PASAT3"**	type 2 diabetes	Pre-diabetic	-4.011	2.26	0.07
		Normal	-7.13	2.35	0.003
	Pre-diabetes	Normal	-3.01	2.34	0.20
**PASAT2"**	type 2 diabetes	Pre-diabetic	-2.30	1.95	0.24
		Normal	-5.40	2.03	0.009
	Pre-diabetes	Normal	-3.09	2.02	0.12
**Symbol Coding**	type 2 diabetes	Pre-diabetic	-7.39	2.16	0.001
		Normal	-11.44	2.24	0.0001
	Pre-diabetes	Normal	-4.04	2.22	0.07

**Figure 1:**
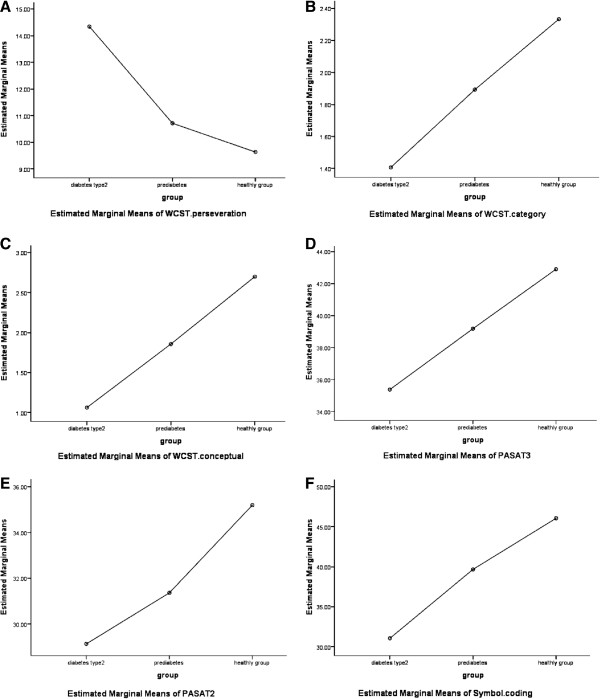
**Estimate margin of executive functioning.** A: WCST (perseveration), B: WCST (category) and C: WCST (conceptual responses), and Estimate margin of information processing (D: pasat3″, E: pasat 2, F: symbol coding, in patient type 2 diabetes, pre-diabetes and control group.

As seen in Figure 1, the mean- adjusted indicated that the score of control group in executive functions and information processing assessments are significantly more and higher than both diabetics and pre-diabetic patients.

## Discussion

The results of this study show that there was a significant difference among normal, diabetic and pre-diabetic groups in executive function of WCST (perseveration) and information processing. In other word the performance of diabetic group was lower than the other two groups. Wateriet et al. [26] in their studies report that there was a significant difference between diabetic and control group in their information processing and executive functions, which is similar to the result of this study. This finding is concordant with the reports of Berg et al. [16] and Elderen et al. [2]. Roriz et al. [19] in a review article concluded that type 2 diabetes is strongly associated with functioning of the brain and diabetes type 2 increases memory deficit and reduces executive functions and information processing. As some recent researches have shown, there is a possibility of a relationship between Alzheimer disease and diabetes [2], and even between pre-diabetics and Alzheimer, as well [18]. Baker et al. [13] reported that there might be a relationship between insulin resistance (per-diabetic period) and deterioration of an Alzheimer type. One of the important purposes of this study was to determine whether pre-diabetic patients show a significant difference in information processing and executive functions, in comparison to normal group? There was no difference among them in the information processing and executive functioning. Ruis et al. [18] in their research reported that patients at early stage of diabetes type 2 becomes significantly worse on cognitive functioning such as information processing, attention and executive functioning, but the mean differences between the group were small, and other research conducted on pre-diabetic patients showed that a pre-diabetic patient becomes worse in memory functioning than normal subjects [10]. The results of our study have shown that there was no difference in information processing and executive functioning in per-diabetic patients in comparison to healthy group, but there was a significant difference in information processing and executive functions in patient with type 2 diabetes. The similarity between our results and other researches ‘again emphasize on the cognitive deficits which are evident in diabetic patients. Another finding of this study was that there is a significant relationship between HbA1c and FBS and some demographics and with WCST (perseveration) in diabetes, which is similar to Yaffe et al. [27]. Our research is a cross- sectional and not longitudinal study; in hence the duration of disease has not been studied. In some studies, possible mild cognitive deterioration have been shown in pre-diabetic stages as revealed in Ruis et al. [18] and Nazaribadie et al. [10] in other cognitive performances such as memory and executive functions. People with impaired glucose tolerance -- the precursor to Type 2 diabetes -- often show impaired cognitive function that may be alleviated through a diet designed specifically for their condition. Impaired glucose tolerance is a pre-diabetic state of hyperglycemia that is associated with insulin resistance and a higher risk of cardiovascular disease. It can precede Type 2 diabetes by several years, and some lifestyle changes, such as getting to a healthy weight and increasing exercise, can help pre-diabetic people avoid that progression completely.

In summary, cognitive decrement can be found in diabetic stage. This finding may have implication for diabetic education and self management in diabetic patients. All of these studies show that the possible link between executive deterioration and diabetes might be serious, and future researches should open a new way in our understanding of the possible related factors.

The prominent innovation of this study was that it was carried out on a sample of pre diabetic patients for the first time. Several limitations of the present study should be considered. First, our study did not analyze the effects of duration of diabetes type 2 and pre-diabetes.The second limitation of this study was that the level of literacy of the patients was supposed to be more than second year of secondary school, however, the literacy of some patients were lower than that. A third limitation was that, Executive functions are very complex cognitive domain, and our tools (wisconsin card sorting test) only measure abstraction and set shifting but this test is a gold standard in measuring this fields and widely used around the worlds.

## Conclusion

In conclusion, these findings suggest that diabetic patients experience decline in executive functioning. Thus, monitoring neuropsychological status besides controlling levels of blood sugar in these patients is important. In this study, some aspects of cognition in patients with diabetes were reviewed. To fully explore the cognitive impairments in people with diabetes, Further studies are recommended.

## Competing interests

The authors report no conflicts of interest. The authors alone are responsible for the content and writing of the article.

## Authors’ contributions

MN, KA and MAM contributed the study design, protocole writing, cell preparation, data collection, analysis interpretations, writing and reviewing of the manuscript. MAH contributed to the interpretation, writing and reviewing the manuscript. SN and SJ had contributed in writing, data collection and cell preparation. All authors read and approved the final manuscript.
